# Fluorescent fullerene nanoparticle-based lateral flow immunochromatographic assay for rapid quantitative detection of C-reactive protein

**DOI:** 10.1186/s40580-019-0207-0

**Published:** 2019-11-01

**Authors:** Kyung Mi Park, Da Jung Chung, Mijin Choi, Taejoon Kang, Jinyoung Jeong

**Affiliations:** 1 BioNano Health Guard Research Center, Korea Research Institute of Bioscience and Biotechnology, Daejeon, 34141 Republic of Korea; 2 Bionanotechnology Research Center, Korea Research Institute of Bioscience and Biotechnology, Daejeon, 34141 Republic of Korea; 3 Department of Nanobiotechnology, KRIBB School of Biotechnology, University of Science & Technology, Daejeon, 34113 Republic of Korea; 4 Environmental Disease Research Center, Korea Research Institute of Bioscience and Biotechnology, Daejeon, 34141 Republic of Korea

**Keywords:** Fullerene, Nanoparticle, C-reactive protein, Lateral flow immunochromatographic assay, Fluorescence

## Abstract

A fluorescent fullerene nanoparticle (NP)-based lateral flow immunochromatographic assay (LFIA) was developed for the rapid and quantitative detection of C-reactive protein (CRP) in serum. The polyclonal CRP-antibody-conjugated fullerene NPs were simply prepared by 1-ethyl-3-(3-dimethyllaminopropyl)-carbodiimide hydrochloride coupling after carboxylation of fluorescent fullerene NPs. By applying the CRP-antibody-conjugated fullerene NPs to a lateral flow test strip, quantitative analysis of CRP in serum was possible at a concentration range of 0.1–10 ng/ml within 15 min. We anticipate that this novel fluorescent fullerene NP-based LFIA can be useful for the rapid and accurate sensing of biological and chemical species, contributing to the disease diagnosis and prognosis, environmental monitoring, and food safety.

## Introduction

The lateral flow immunochromatographic assay (LFIA) is a common technique for the detection of such diverse analytes as hormones, disease-related biomarkers, and toxins in the clinical, environmental, and food industry fields, because of its simplicity and rapidity [1–8]. As a standard reporting material in LFIA, colloidal gold (CG) has been widely used for colorimetric detection due to its visibility. However, the CG-based LFIA often suffers from limitations such as lack of sensitivity and the ability to provide only qualitative/semi-quantitative analysis. To overcome the drawbacks of CG-based LFIA, various materials have been developed as reporters, including fluorescent microspheres (FMs), quantum dots (QDs), up-conversion nanoparticles (UCNPs), carbon nanoparticles (CNPs), and platinum nanoparticles (PtNPs) [9–13]. Although these reporter materials have enabled sensitive and quantitative analyses of molecules even at low analyte concentrations, challenges remain in terms of material preparation, functionalization of the materials for efficient conjugation of the target molecules, and optimization of sensing conditions on a lateral flow assay. Previously, we developed a novel fluorescent fullerene material, tetraethylene glycol-conjugated fullerene nanoparticles (C_60_-TEG), that was prepared via a simple procedure involving lithium hydroxide as a catalyst at room temperature [14, 15]. These fluorescent fullerene nanoparticles (NPs) are easy to prepare compared to other inorganic materials, i.e., QDs and UCNPs, that require large amounts of surfactants, complex purification steps, and harsh conditions such as high temperatures for synthesis. Furthermore, the fullerene NPs can provide distinct and controllable fluorescent signals. These unique properties of C_60_-TEG prompted us to employ them for LFIA.

Herein, we report a new fluorescent probe (C_60_-TEG)-based LFIA, for the highly sensitive, rapid, and quantitative analysis of C-reactive protein (CRP) in serum. CRP is known as an acute-phase plasma protein that is a non-specific but sensitive inflammation marker, especially in the case of bacterial infection. It is also known as a potential indicator of cardiovascular disease, e.g., coronary heart disease, ischemic stroke, and acute myocardial infarction [16–18]. Because the measurement of low concentrations of CRP is critical for early diagnosis of inflammation and cardiovascular disease, many researchers have attempted to develop a highly sensitive CRP-detectable LFIA [19–21]. For example, Swanson et al. recently reported a CRP detection limit of 10 ng/ml using near-infrared dye-LFIA [22]. In this work, we demonstrated the quantitative analysis of CRP in the presence of serum with a wide dynamic range of 0.1–10 ng/ml by using the polyclonal anti-CRP-conjugated C_60_-TEG (pAb-CRP-C_60_-TEG) as a fluorescent probe. The pAb-CRP-C_60_-TEG was simply prepared by 1-ethyl-3-(3-dimethyllaminopropyl)-carbodiimide hydrochloride (EDC) coupling after carboxylation of fluorescent fullerene NPs. Since the developed C_60_-TEG-based LFIA achieves sufficiently high sensitivity and quantitative analysis of a target molecule, the C_60_-TEG-based LFIA can be used as an advanced fluorescent LFIA for disease diagnosis and prognosis, environmental monitoring, and food safety.

## Results and discussion

### Synthesis and characterization of pAb-CRP-C_60_-TEG

**Fig. 1:**
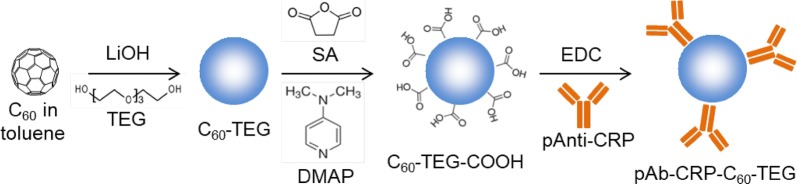
Schematic illustration of synthetic procedure of pAb-CRP-C_60_-TEG for LFIA

Figure 1 is synthetic procedure of pAb-CRP-C_60_-TEG for LFIA. Firstly, the C_60_-TEG was prepared by adding LiOH to a mixture of C_60_ and TEG. Then, the NPs were modified to expose a carboxylate group by the reaction with SA and DMAP. Next, the C_60_-TEG-COOH and pAb-CRP were conjugated via EDC coupling (Fig. 1). This pAb-CRP-C_60_-TEG preparation process is uncomplicated and easy to perform under ambient conditions, whereas other reporting materials, e.g., semiconducting QDs and UCNPs, are synthesized at high temperatures and in organic solvents. In addition, the C_60_-TEG-COOH does not need to be water-soluble intentionally because it is highly hydrophilic.

**Fig. 2:**
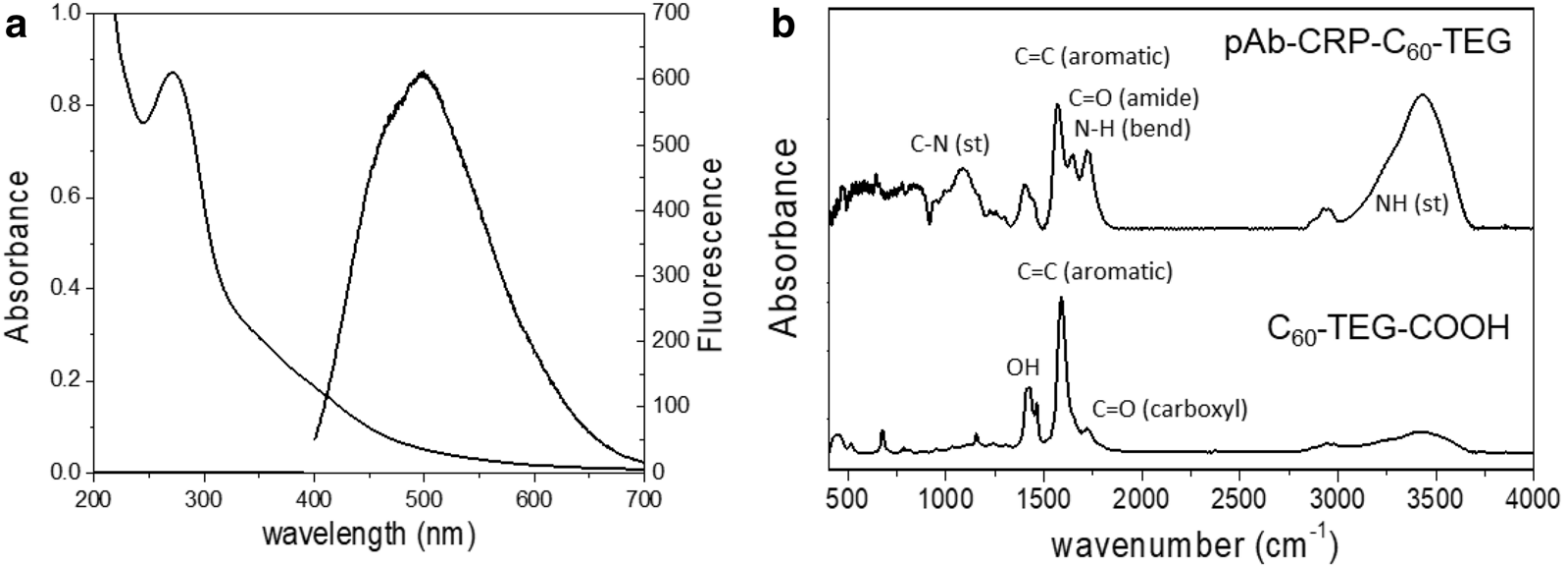
**a** Absorption and fluorescence spectra of C_60_-TEG-COOH. **b** Infrared spectra of C_60_-TEG-COOH and pAb-CRP-C_60_-TEG

The absorption and fluorescence spectra of the C_60_-TEG-COOH are shown in Fig. 2a. The absorption spectrum shows a strong peak at 272 nm and broad bands at 300–400 nm, indicating the results from carboxylation of C_60_-TEG which has weak broad band at 260 nm and 350 nm [14]. These features are similar to those of the spectra of the hydrophilic fullerene carboxylic acid derivative and fullerenol, respectively [23, 24]. In addition, the fluorescence spectrum of C_60_-TEG-COOH exhibits broad fluorescence with a maximum peak at 500 nm under excitation at 350 nm, which is slightly blue-shifted compared to the fluorescence ofC_60_-TEG, meaning that the carboxylation may lead to change of the optical properties from C_60_-TEG. However, the fluorescence quantum efficiency of C_60_-TEG-COOH (Φ_F_ = 0.33) was higher than that of C_60_-TEG (Φ_F_ = 0.095) so that it is sufficient to use as a fluorescent probe for LFIA.

After conjugating pAb-CRP with C_60_-TEG-COOH via an EDC coupling reaction, we used infrared spectroscopy to examine the antibody conjugation with the C_60_-TEG-COOH. The infrared spectra in Fig. 2b reveal that the C_60_-TEG-COOH was successfully conjugated with pAb-CRP by the formation of amide bond (C=O amide at 1645 cm^−1^ and N–H stretch at 3430 cm^−1^). C–N stretch at 1081 cm^−1^ shows the presence of amine bond from the antibody. In comparison, the simple carboxylic acid peaks appeared at 1425 cm^−1^ (OH bending) and 1716 cm^−1^ (C=O stretching) in the spectrum of unconjugated C_60_-TEG-COOH. We also determined, via the Bradford assay, that the amount of conjugated pAb-CRP was 4.58 µg/mg of C_60_-TEG-COOH.

### Preparation and optimization of C_60_-TEG-based LFIA

**Fig. 3:**
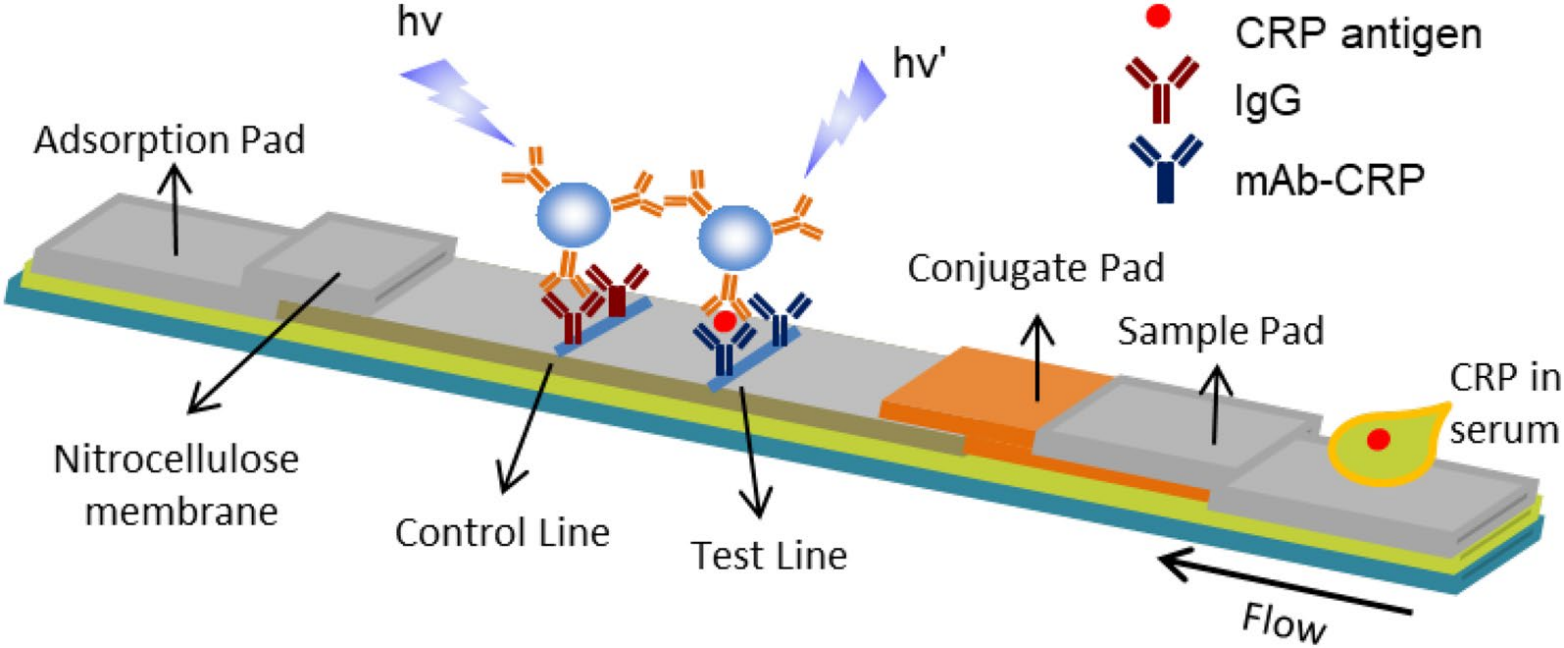
Schematic diagram of C_60_-TEG-based LFIA for the detection of CRP

By using the pAb-CRP-C_60_-TEG, we designed the fluorescent immunochromatographic assay on a lateral flow strip for the rapid and quantitative detection of CRP (Fig. 3). First, the pAb-CRP-C_60_-TEG was dispensed on the conjugate pad of a lateral flow test strip. The anti-mouse IgG and mAb-CRP were placed on the nitrocellulose membrane using a dispenser, forming the CL and TL, respectively. For the detection of CRP, the sample solution was applied onto the sample pad. Then, the solution migrated to the conjugate pad, where the CRP can bound with pAb CRP-C_60_-TEG. Next, free pAb-CRP-C_60_-TEG and CRP-binding pAb-CRP-C_60_-TEG were captured by anti-IgG in the CL and mAb-CRP in the TL, respectively. After 15 min of migration, the strip was subjected to a fluorescence measurement system for the determination of the TL/CL fluorescence signal ratio.

We optimized the type of blocking solution and its dilution factors to reduce the non-specific adsorption of pAb-CRP-C_60_-TEG during flow through the strip. After testing three different blocking solutions, each containing casein, skim milk, and BSA in up to tenfold dilutions, we found that 1% BSA solution was the most efficient in obtaining a fluorescence response with a high signal-to-noise ratio. Moreover, other parameters, e.g., the volume of sample solution, the washing steps with PBS to remove the unbound fluorescent conjugates, and the immunoreaction time, were also optimized to increase the sensitivity.

### Quantitative detection of CRP using C_60_-TEG-based LFIA

**Fig. 4:**
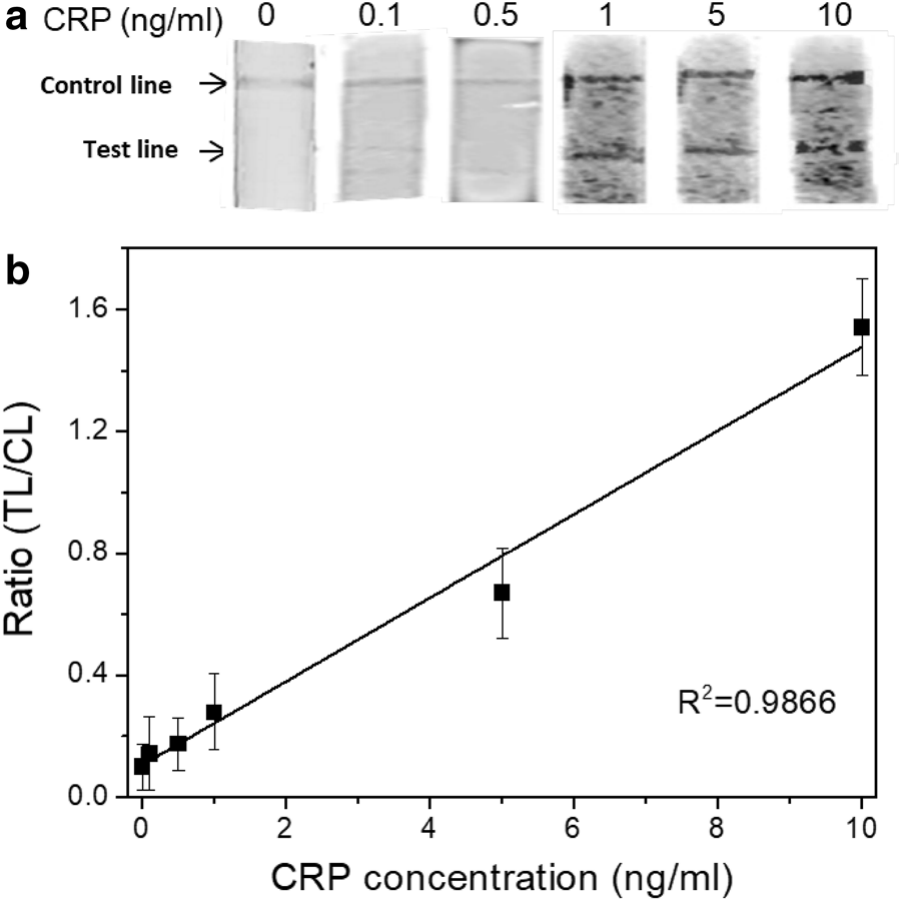
**a** The fluorescence images of the test strips with various concentration of CRP from 0.01 to 10 ng/ml. **b** The calibration curve obtained from the area ratio (TL/CL) against the concentration of CRP in serum

On the basis of the optimal experiment conditions, the analytical performance for quantitative measurement of CRP was further evaluated with standard CRP samples in serum. Figure 4a shows fluorescence images of the TLs and CLs on the test strip with various CRP concentrations as high as 10 ng/ml. In the absence of CRP, the CL was easily observed, whereas the TL was not observed. Both the TL and the CL were visible in the presence of CRP, and the lines became more vivid as the CRP concentration was increased, indicating that additional pAb-CRP-C_60_-TEG was captured on the TL. The fluorescence signal ratio of the TL and CL, as analyzed by image analysis software, is shown in Fig. 4b. The relative ratio of TL/CL linearly increases with increasing CRP concentration in the range of 0.1–10 ng/ml. These results are comparable with those of previous studies on the detection of CRP using various methods, including commercial- and microfluidic-based point-of-care immunodiagnostics [25, 26].

Immunochromatographic strips offer benefits because of their user-friendly format, short test times, long-term stability, and relatively low fabrication costs. Although CG-based strip sensors are a standard immunochromatographic method, this method suffers from limitations such as low sensitivity and color interference in hemolytic samples. However, the fluorescent LFIA provides advantages such as high sensitivity, quantitative measurement, and lack of color interference. In this study, fluorescent fullerene nanoparticles (C_60_-TEG) were used as a new reporting material in a fluorescent LFIA. The C_60_-TEG can be simply prepared and modified to conjugate antibodies and is adaptable in a nitrocellulose strip because of its hydrophilicity and relatively small size, which may facilitate flow through the membrane.

## Conclusion

A new fluorescent LFIA using C_60_-TEG was developed for the detection of a wide range of CRP concentrations. The C_60_-TEG-COOH was conjugated with pAb-CRP via an EDC reaction, and the conjugates were used as a fluorescent probe as they migrated from the conjugate pad to the CL and TL, which were printed with anti-mouse IgG and mAb-CRP in a strip, respectively. The TL/CL fluorescence signal ratio increased as the CRP concentration was increased from 0.1 to 10 ng/ml in serum. The fluorescent fullerene nanoparticle-based LFIA was simply prepared and successfully used to detect a wide range of CRP concentrations. Therefore, the combined fluorescent fullerene nanoparticle-based LFIA exhibits strong potential for highly sensitive, rapid, and quantitative immunoassays.

## Experimental section

### Materials and chemicals

Buckminsterfullerene (C_60_, 99.9%) was purchased from SES Res. (TX, USA). Toluene (99.8%), tetraethylene glycol (TEG), lithium hydroxide (LiOH), dimethyl sulfoxide (DMSO), succinic anhydride (SA), 4-(dimethylamino)pyridine (DMAP), diethyl ether (DE), EDC, anti-mouse immunoglobulin G (anti-mouse IgG), and bovine serum albumin (BSA) were purchased from Sigma-Aldrich (St. Louis, MO, USA). Ethyl acetate (EA, 99%) was obtained from Daejung (Seoul, Korea). Ethanol was obtained from Merck (Darmstadt, Germany). Dulbecco’s phosphate-buffered saline (PBS) was obtained from Gibco (NY, USA). CRP and polyclonal CRP antibody (pAb-CRP) were purchased from Abcam. Monoclonal CRP antibody (mAb-CRP) was obtained from R&D Systems. All chemicals were used without further purification.

### Synthesis and characterization of C_60_-TEG-COOH

The fluorescent fullerene NPs were synthesized using a modified version of a process reported in the literature [14]. First, C_60_ solution (10 ml in toluene) at a concentration of 0.25 mg/ml was added to 10 ml of TEG. Next, LiOH (40 mg) was added to the mixture of C_60_ and TEG, turning the color of solution from pink to dark-brown within 10 min. After stirring of the solution for 20 h, the resultant fullerene NPs were precipitated by the addition of excess EA. The precipitates were collected by filtration and dried to obtain C_60_-TEG powders. To conjugate the anti-CRP, the C_60_-TEG was firstly functionalized to expose carboxylic acid groups on the surface of the NPs. 10 mg of C_60_-TEG powder was dissolved in 1 ml of DMSO, and 50 mg of SA and 15 mg DMAP were added to the C_60_-TEG solution. After the solution was stirred for 20 h, the resultant NPs were precipitated by the addition of excess DE. The precipitates were collected by filtration and dried to obtain carboxylated C_60_-TEG (C_60_-TEG-COOH) powder. The optical and chemical properties of C_60_-TEG-COOH were analyzed by UV/Visible spectroscopy (Beckman Coulter, DU-800, USA), fluorescence spectroscopy (Perkin-Elmer, LS55, UK), and FTIR spectrophotometry (Bruker Optics, IF66, USA) using the KBR-pellet method.

### Preparation of pAb-CRP-C_60_-TEG

The pAb-CRP-C_60_-TEG was prepared by an EDC coupling reaction between C_60_-TEG-COOH and pAb-CRP. The C_60_-TEG-COOH was dissolved in PBS at a concentration of 10 mg/ml, and 10 µl of 0.1 M EDC and 10 µl of 0.1 mg/ml pAb-CRP were added to the 100 µl of C_60_-TEG-COOH solution. The reaction mixture was maintained at room temperature for 1 h. The resulting mixture (pAb-CRP-C_60_-TEG) was purified by spin chromatography using a PD Spin Trap G-25 column (GE Healthcare) and collected as eluates after centrifugation of the column at 2500 rpm for 2 min. Finally, 1% BSA (w/v) solution was further added to the eluted solution for blocking.

### Preparation of a lateral flow immunochromatographic test strip

The test strip involved (i) a sample pad, (ii) a nitrocellulose (NC) membrane, and (iii) an absorption pad, and all attached to a backing card. The test zone involved immobilized mAb-CRP (0.5 mg/ml in PBS) as the test line (TL) and anti-mouse IgG (1 mg/ml in PBS) as the control line (CL), both of which were dispensed at 1 µl/cm using an automatic dispenser. After drying for 1 h at 37 °C in an incubator, the membranes were reacted with a blocking solution (1 mg/ml BSA in PBS) for 30 min at 20 °C. After the absorption pad and the sample pad were affixed to the top and the bottom of the membrane, respectively, the membranes were cut into strips 3–4 mm wide using an automatic programmable cutter (GCI-800, Guillotine Cutting, ZETA Corporation, Korea). The diluted pAb-CRP-C_60_-TEG was dispensed onto the conjugating pad prior to the plate being pasted on with an overlap of 2 mm with the NC membrane. The sample pad was pasted onto the same end with its margin justified to the conjugating pad. Finally, after being sealed, the test strip was stored in a desiccator until use.

### Detection of CRP using C_60_-TEG-based LFIA

Various concentrations of CRP solution were prepared in human serum solution. The LFIA strips were dipped into 96-well plate wells that contained CRP solution (0.1 ml). After 15 min, the fluorescence signals of both the CL and the TL on the strips were measured using a luminescence image analyzer (LAS-3000, FujiFilm) and excitation and emission wavelengths of 460 and 510 nm, respectively. The fluorescence intensities of the TL and CL were analyzed from a camera image using the MultiGauge 3.0 software.

## Data Availability

All data generated or analysed during this study are included in this published article.
